# High Internal-Phase Pickering Emulsions Stabilized by Xanthan Gum/Lysozyme Nanoparticles: Rheological and Microstructural Perspective

**DOI:** 10.3389/fnut.2021.744234

**Published:** 2022-01-05

**Authors:** Wei Xu, Zhifan Li, Haomin Sun, Shuqing Zheng, He Li, Denglin Luo, Yingying Li, Mengyuan Wang, Yuntao Wang

**Affiliations:** ^1^College of Life Science, Xinyang Normal University, Xinyang, China; ^2^Tea Plant Biology Key Laboratory of Henan Province, Xinyang Normal University, Xinyang, China; ^3^College of Food and Bioengineering, Henan University of Science and Technology, Luoyang, China; ^4^College of Food and Bioengineering, Zhengzhou University of Light Industry, Zhengzhou, China

**Keywords:** xanthan gum, lysozyme, nanoparticles, high internal phase, Pickering emulsion

## Abstract

Food-grade high internal-phase Pickering emulsions (HIPPEs) stabilized by solid or colloidal particles with different advantages have attracted extensive attention nowadays. However, looking for new appropriate particle stabilizers is the common practice for HIPPEs preparation. It is crucial to find a new strategy for the development of functional HIPPEs with controllable properties. In this study, a high concentration of xanthan gum/lysozyme nanoparticles (XG/Ly NPs) was used for the preparation of HIPPEs for the first time. Visual observations, creaming index (CI), microstructure, and rheology tests were carried out to investigate the potential of XG/Ly NPs as HIPPEs stabilizers. Results indicated that XG/Ly NPs could stabilize oil droplets in the concentration range of 0.5–4% (w/v). The HIPPEs with a minimal particle concentration of 1% exhibited remarkable physical stability. Rheological measurements showed that a high stability of solid-like HIPPEs was successfully obtained with a higher concentration of XG/Ly NPs. Overall, the HIPPEs stabilized by different concentrations of XG/Ly NPs exhibited excellent rheological and structural properties, which might provide a feasible strategy for the development of functional emulsion systems with controllable structures.

## Introduction

High internal-phase Pickering emulsions are emulsions with an internal-phase ratio higher than 0.74. They are widely used in food, cosmetics, tissue repair, medicine, and the petroleum industry, and other fields due to their high oil loading capacity and tunable viscoelasticity (1–3). Compared with water-in-oil (W/O) emulsions, oil-in-water (O/W) emulsions, high internal-phase Pickering emulsions (HIPPEs) generally exhibit a higher stability because of the low mobility of oil droplets than water droplets, and they can be easier to prevent the occurrence of sedimentation, flocculation, or coalescence than W/O emulsions (4). Usually, the oil droplets in HIPPEs are arranged in a polydisperse arrangement or deformed into a polyhedron arrangement, while the continuous phase appears as a thin film that separates the dispersed phase which could improve the space utilization of the total volume of the emulsion (5, 6). The traditional HIPEs stabilized by inorganic particles or even surfactants are less biocompatible because they may contain potentially toxic polymer components. The surfactants were generally used in a large quantity to prepare the HIPPEs, and the inorganic particles cannot stabilize HIPEs without chemical modification (4). These results inevitably cause the consumers' concerns about their safety (7). However, HIPPEs stabilized by solid or colloid particles have strong adhesive and can be irreversibly adsorbed on the oil-water interface, providing the emulsions shelf-life for months or even years (8). Therefore, new natural and non-toxic stabilizers are valued in the preparation of HIPPEs, and they are expected to replace the traditional surfactant-stabilized HIPEs in the food industry (9).

In recent years, studies have reported that stable HIPPEs could be obtained using various particles as stabilizers, such as stable Pickering HIPEs stabilized by gliadin/chitosan complex particles (10), pH-Sensitive W/O Pickering HIPEs, and W/O/W double HIPEs stabilized by lecithin and silica inorganic particles (4), HIPEs stabilized by food-grade starch (11), edible HIPPEs with double-emulsion morphology stabilized by zein protein nanoparticles (12) and novel antioxidative HIPPE stabilized by tea polyphenol nanoparticles (7). To our knowledge, xanthan gum/lysozyme nanoparticles (XG/Ly NPs) could use as emulsifiers and stabilize Pickering emulsion. However, XG/Ly Nps act as emulsifiers in HIPPEs have not been further researched (13).

Tea oil is a kind of advanced edible oil extracted or pressed from the fruit of *Camellia oleifera*. It contains high nutritional and health functions, which are of great significance for the prevention and treatment of hypertension and cardiovascular diseases (14). Nevertheless, many unsaturated fatty acids presented in tea oil causes different degrees of fatty acid failure under air, light, humidity, and high temperature, which could reduce the nutritional value and sensory acceptability of the tea oil, and lead to the formation of toxic substances (15). The HIPPEs formed by solid or colloidal particles as stabilizers could encapsulate liquids or other unstable molecules that are sensitive to the external environment (oxygen, light, and heat), which may have a protective effect on volatile and oxidized bioactive ingredients (7, 16). However, there is no systematic study on HIPEs using tea oil.

According to previous studies, the microstructure and viscoelastic of Pickering emulsions could be adjusted by the variation of particle concentration (17). The appropriate increase in particle concentration could increase the viscosity of the emulsion systems, which could effectively inhibit the gravitational separation of the dispersed and continuous phases in the emulsions and reduce the emulsification speed. The stability of the Pickering emulsions could be improved by the formation of a thick particle adsorption layer at the interface between the continuous phase and the dispersed phase (18). For example, a weak particle network exists in the aqueous continuous phase when the concentration of zein/tannic acid complex particles was lower than 2% (w/v). While when the particle concentration increases to 5%, the droplet size of the emulsion is significantly reduced, and the gel strength of the emulsion was improved by the particle network forming in the continuous phase (17).

Based on the above considerations, the main objective of the present work was to investigate the effectiveness of self-assembled XG/Ly NPs as a stabilizer for HIPPEs, and further explore the characteristics of HIPPEs with the rich nutritional value of tea oil as the oil phase to facilitate utilization of *Camellia oleifera* resources to a greater extent.

In this article, different concentration of xanthan gum/lysozyme nanoparticles (XG/Ly NPs) was used for the preparation of HIPPEs for the first time. Visual observations, creaming index, microstructure, and rheology properties of HIPPEs were investigated.

The endeavor might provide a feasible strategy for the development of functional HIPPEs systems with controllable respective structures and properties.

## Materials and Methods

### Materials

Xanthan gum (XG) (Mw = 1.3 × 10^7^ Da) and Nile Blue A were purchased from Shanghai Yuanye Bio-Technology Co., Ltd (China). Ly (Mw = 14.3 kDa) from chicken egg white and Nile Red were provided by Sinopharm Chemical Reagent Co., Ltd (China). Food-grade tea oil was bought from a local supermarket (Xinyang, Henan Province, China). All the solutions in the experiments were prepared using ultrapure water through a Milli-Q water purification system (Millipore, Milford, MA, USA). All the solutions in the experiments were prepared with ultra-pure water purified by the Milli-Q system.

### Preparation of XG/Ly NPs

Xanthan gum/lysozyme nanoparticles (XG/Ly NPs) were prepared using the previous method (13). In brief, XG and Ly solutions (1 mg/ml) were stirred with ultrapure water at room temperature for 6 and 2 h, respectively. XG/Ly mixture with a weight ratio of 1:1 was prepared after pH was adjusted to 11.8. The mixtures were further heated at 80°C for 15 min and cooled to room temperature spontaneously. Before 24 h of dialysis, the pH of the XG/Ly NPs solution was regulated to 7 by using 1 M HCl. The obtained XG/Ly NPs solution was filtered *via* an 80 μm membrane to remove impurities prior to further use. And then the XG/Ly NPs solutions were freeze-drying and collected.

### Preparation of HIPPEs

High internal-phase Pickering emulsions (HIPPEs) were prepared using a one-step emulsification method in accordance with the method of Yang et al. (2). XG/Ly NPs solutions with different particle concentrations (0.5, 1, 2, and 4%, w/v) were used as the aqueous phase. Tea oil was used as an oil phase. First, tea oil and XG/Ly NPs solutions were mixed with a fixed oil content of 80% (v/v). Then, the mixtures were homogenized using an IKA Ultra-Turrax T25 homogenizer (Germany) at 18,000 rpm for 3 min. The freshly prepared HIPPEs were allowed to rest at room temperature (25°C) for 2 h to achieve a stable state before further characterization.

### Microstructure

The micromorphology of the HIPPEs was visualized using optical microscopy (Leica, Germany) with a 40× objective. The images were taken by a microscope. Confocal laser scanning microscopy (clsm, Leica TCS SP8, Germany) was used to observe the microscopic morphology of the HIPPEs to further understand the distribution of the dispersed phase and the continuous phase. The excitation wavelengths of Nile Red and Nile Blue A were 488 and 633 nm, respectively. Before the measurement, each sample was dyed with mixed dye formed by dissolving 0.1% Nile Red and 0.1% Nile Blue A in 1,2-propanediol. Then, 40 μl of mixed dye was added to 1 ml emulsions. All representative images were caught at a magnification of 20× using the stimulated emission depletion (STED) system after 1 h stirring at 25°C. The internal microstructure of the emulsion was characterized by Cryo-SEM (FEI Quanta 450, USA) under high vacuum conditions to explore the distribution of XG/Ly NPs on the oil-water interface.

### Creaming Index

Creaming index (CI) is used to evaluate the stability of the emulsion system and provide information about the development of droplet aggregation (19). In the present study, the value of CI (%) for various HIPPEs was recorded immediately after the emulsifying process. The freshly prepared HIPPEs were placed into a 25 ml graduated cylinder and stored at room temperature (25°C) for 3 h to record the stratification of the emulsions. Three replicates were performed per sample. CI was determined in accordance with the method of previous studies as follows (20):


(1) $$\begin{array}{l} \text{CI}\left(\%\right)=\frac{{\text{H}}_{1}}{{\text{H}}_{2}}\times 100 \end{array}$$

where H_1_ is the height of the separated water layer, and H_2_ is the total height of the HIPPEs.

### Rheological Characterization

All rheological properties of HIPPEs prepared with 80% oil content were investigated using a Discovery HR-2 rheometer (TA Instruments, US) at 20 C using a steel parallel plate (40 mm). The gap between the two plates was adjusted to 1,000 μm. The apparent viscosity as a function of shear rate ranging from 0.1 s^−1^ to 100 s^−1^ was measured. Next, frequency sweeps were performed from 0.01 to 100 Hz with a strain of 1% within the linear viscoelastic region. In addition, amplitude sweeps were carried out with increasing the strain from 0.1 to 100% at a fixed frequency of 1 Hz.

## Results and Discussion

### Microstructural Analysis

**Figure 1:**
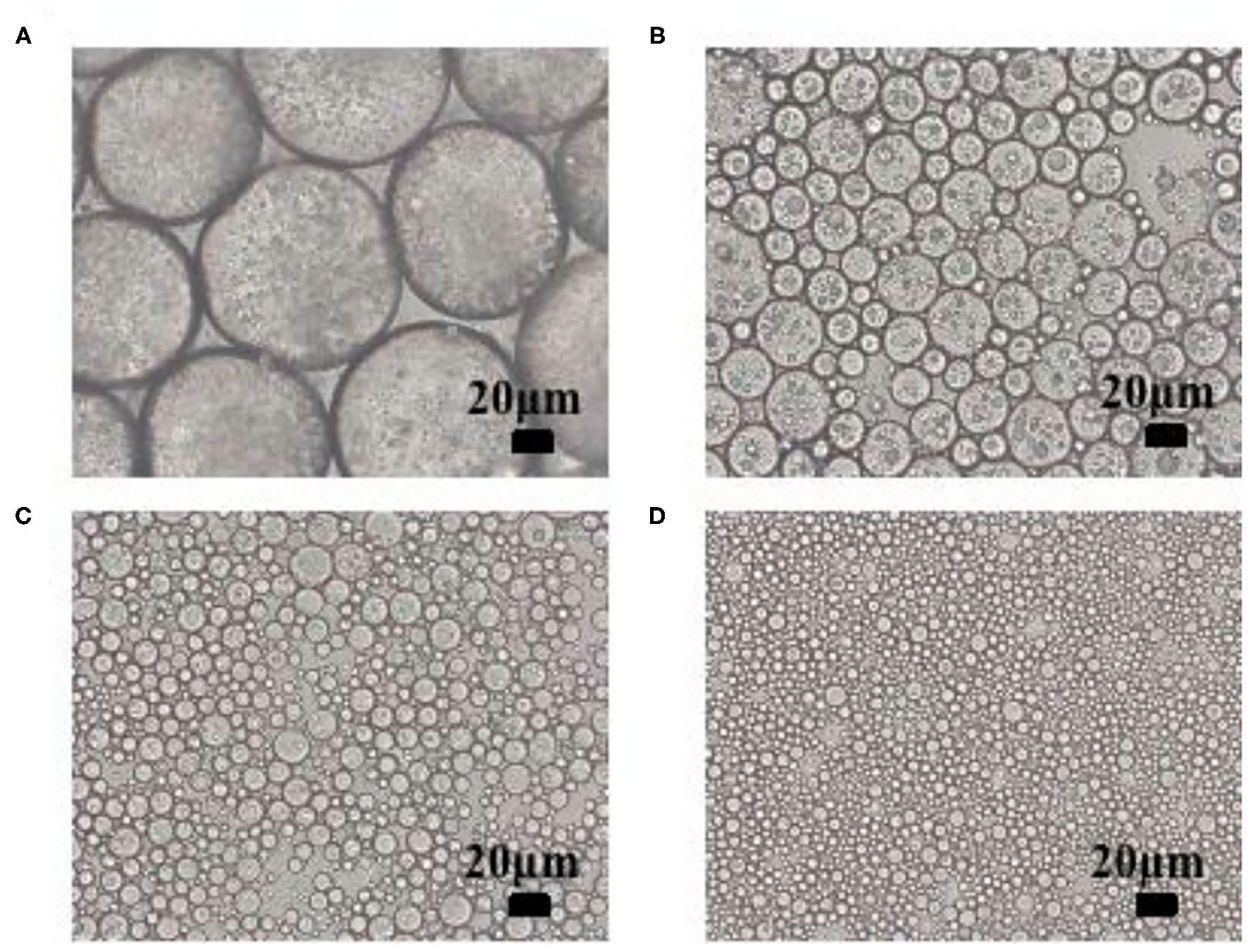
Microstructure of high internal phase Pickering emulsions with various particle concentrations [**(A)** 0.5%, **(B)** 1%, **(C)** 2%, **(D)** 4%].

Figure 1 shows that the droplets of the HIPPEs presented a homogeneous spherical shape, and the droplet sizes were dependent on the concentrations of xg/ly nps. The droplets with 0.5% xg/ly nps concentration were relatively sparse and larger than others, which displayed a decreasing trend with the increase of particle concentration (1–4%). For the emulsion with 4% xg/ly nps, the adjacent droplets were closely arranged or even slightly overlapped. These results were attributed to a solid-like three-dimensional network structure that was formed in the continuous by the excess xg/ly nps, which led to the decrease in the droplet size of the HIPPEs and provided gel properties for the structure (13). The crosslinking within the network may be enhanced with the increase in xg/ly nps concentration, which could make the structure tighter (21).

**Figure 2:**
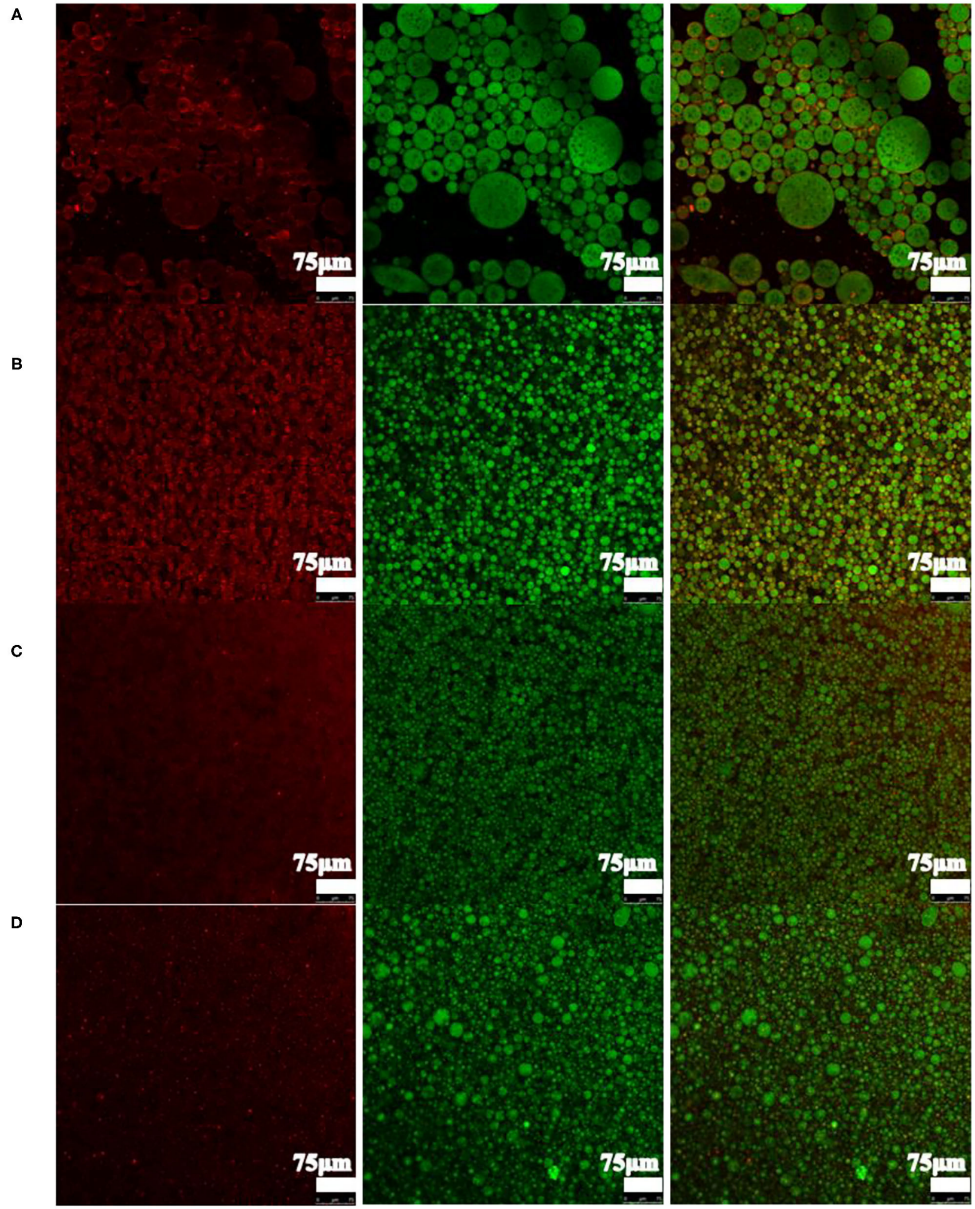
CLSM observation of high internal phase Pickering emulsions with various particle concentrations [**(A)** 0.5%, **(B)** 1%, **(C)** 2%, **(D)** 4%].

To investigate the formation mechanism for the HIPPEs, the microstructure of the HIPPEs made from various particle concentrations (0.5–4%) are analyzed using the CLSM technique and the results are shown in Figure 2. In this work, Nile red was used for dyeing the dispersed phase (excitation at 488 nm, green color), and Nile Blue A used for dyeing the aqueous continuous phase (excitation at 633 nm, red color). CLSM observations suggested that HIPPEs exhibited significant microstructure differences depending on particle concentration. More xg/ly nps distributed in the continuous phase when the particle concentration increased. However, the droplet size decreased gradually with the xg/ly nps concentration increasing from 0.5 to 4%. At low particle concentration (0.5%), the oil droplets were larger than others and distributed in the HIPPEs unevenly (Figure 2A). This discovery is probably attributed to the coalescence phenomenon that occurred between the oil droplets due to the particles being insufficient to encapsulate larger oil droplets. The lower viscosity value of the emulsion (**Figure 5**) was also responsible for this phenomenon. With the particle concentration further increased to 1% or above, smaller emulsion droplets were evenly and tightly dispersed in the aqueous phase, which could generally be preferable for the HIPPEs stability (22). In particular, at 4% particle concentration, the emulsion droplets were closely packed with one another, and the xg/ly nps contributed to the network formation. The formed emulsion was stable, and its firmness was increased by the stronger interactions between the droplets (23). This phenomenon, reflecting the discrepancy in the amount of the XG/Ly NPs absorbed on the oil-water, either interfaces or gets involved in the formation of the gel-like network.

### Creaming Index

**Figure 3:**
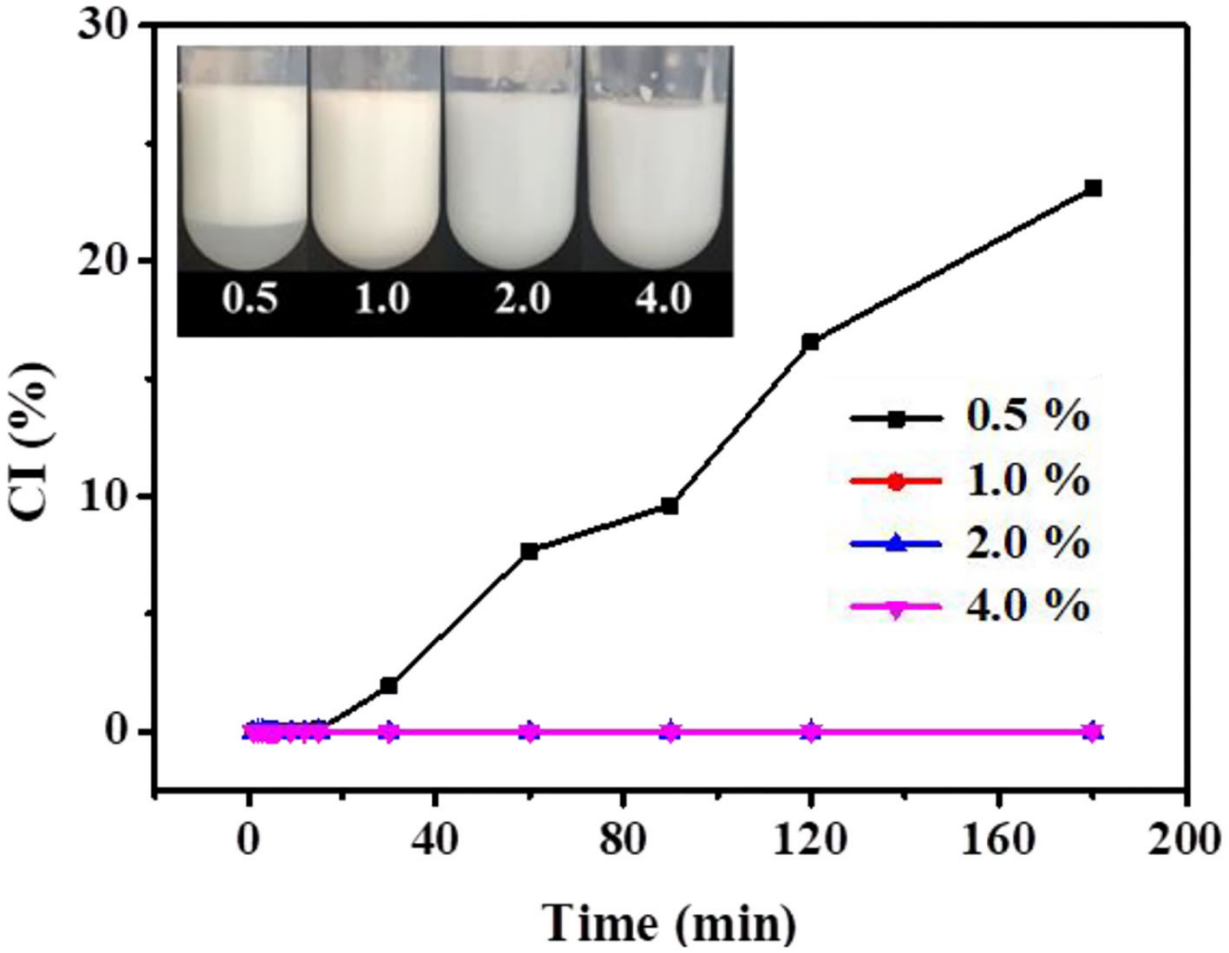
Creaming index of high internal phase Pickering emulsions with various particle concentrations (0.5–4%).

The influence of xg/ly nps concentration on the emulsifying properties of the HIPPEs at 25°C for 3 h is evaluated using CI according to the method of Li et al. (13). As shown in Figure 3, increasing the xg/ly nps concentration lowered the CI for HIPPEs. At lower xg/ly nps concentration (0.5%), creaming increased as the time increased, possibly due to fewer particles are not enough to stabilize a larger volume of oil droplets, causing the internal structure to collapse due to its own gravity. However, the emulsions with xg/ly nps concentrations of 1, 2, and 4% (w/v) do not exhibit any creaming, which can be explained as higher xg/ly nps concentrations increase the density of oil droplets and the emulsion viscosity (19). In addition, the stability of the HIPPEs was improved as the xg/ly nps concentration increased from 0.5 to 4% because of the gradually formed gel-like network in the system. Similar phenomena were also found in corn oil-in-water emulsion prepared with fish gelatin as the stabilizer (19), camellia oil-based W/O emulsions stabilized by tea polyphenol palmitate (20), and Pickering HIPEs stabilized by gliadin/chitosan complex particles (10).

### Visual Observations

**Figure 4:**
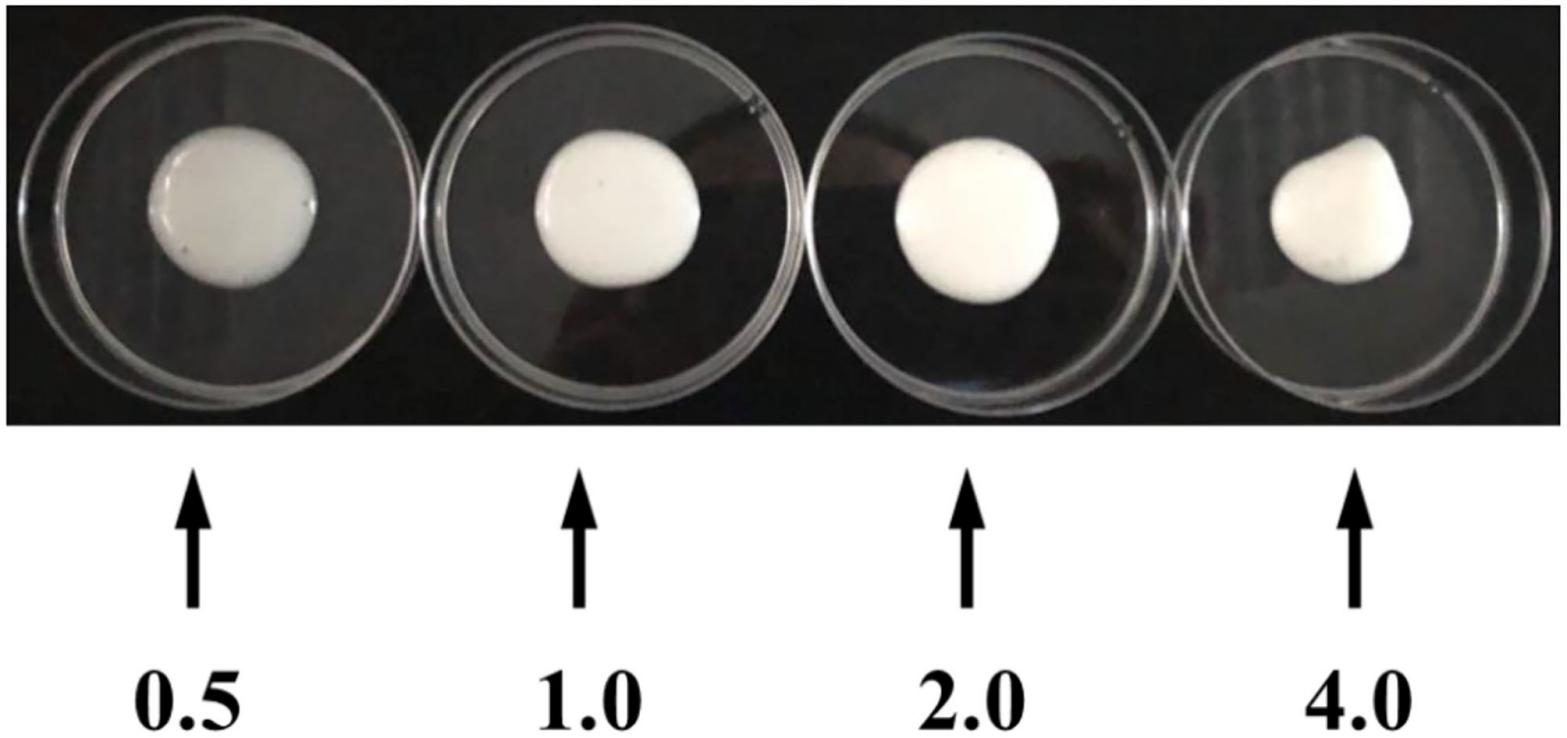
Visual images of high internal phase Pickering emulsions at various XG/Ly NPs concentrations (0.5–4%) after storage of 2 h at room temperature, obtained by homogeneous.

Figure 4 shows visual observations of homogeneous emulsions at 0.5–4% particle concentration with 80% oil content after a 2-h storage period (25°C). Differently, the emulsion with 0.5% xg/ly nps had a slightly transparent appearance compared to others. According to previous research, we speculated that aggregation and flocculation are generally formed, and there were likely three different separated phases (water, emulsion, and oil phases) in this system, which were attributed to the insufficient of xg/ly nps for complete coverage of tea oil and little steric or repulsion between droplets cannot prevent droplet collision (19). This result was also consistent with the result of its rapid creaming behavior (Figure 3). Interestingly, the HIPPEs made from 4% xg/ly nps displayed a gel-like character because of their self-standing property, whereas those from 0.5 to 2% xg/ly nps remained in a flowing liquid. In the latter case, thicker emulsions were observed at a higher particle concentration, whereas in the former case, elasticity emulsions with more elasticity and firmness were formed at 4% particle concentration. A similar observation has been reported for emulsions stabilized by whey protein concentrate (23).

### Apparent Viscosity

**Figure 5:**
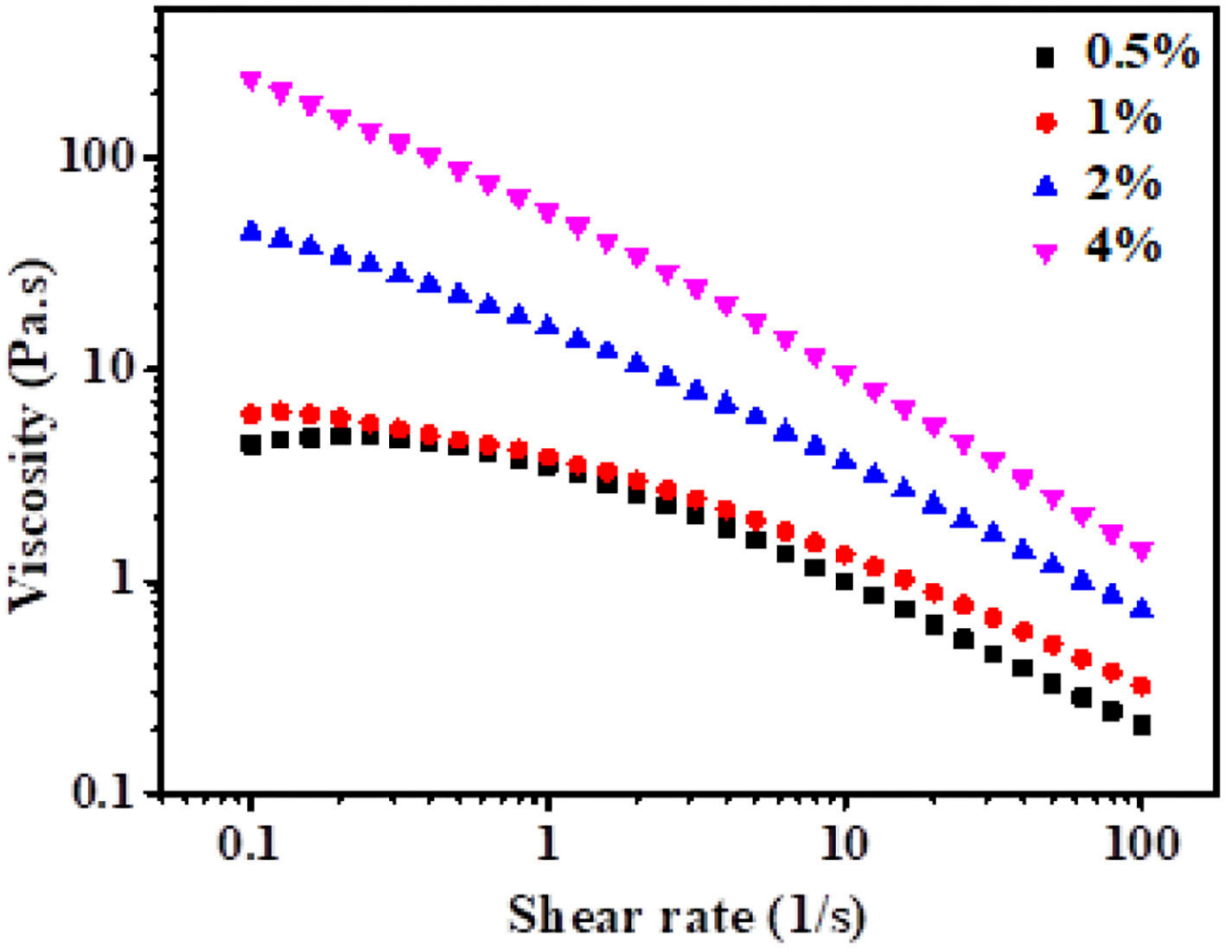
The viscosity of high internal phase Pickering emulsions with various particle concentrations (0.5–4%).

The apparent viscosity of the HIPPEs stabilized by xg/ly nps is depicted in Figure 5. Shear-thinning behavior was presented as the apparent viscosity decreased along with the increase in the shear rate for all samples. This finding is related to the arrangement of xg/ly nps along the shear direction and the decrease in the initial band between the nanoparticles (24). The shear-thinning behavior of the emulsion stabilized by the xg/ly nps system has also been reported in previous literature (13). The apparent viscosity of HIPPEs increases as the xg/ly nps concentration increases at a given shear rate. Furthermore, the apparent viscosity is dependent on the shear rates. In the same shear rates, the sample with 4% xg/ly nps showed higher apparent viscosity. After 4% xg/ly nps was introduced, the apparent viscosity shifted from 4.87 to 238.17 Pa·s at 0.1 s^−1^. A similar trend was found in the emulsions stabilized by chitin particle/tannic acid complex and sodium caseinate/inulin/konjac glucomannan (25, 26). With the increase in particle concentration, the agglomeration on the surface of oil droplets is one of the reasons for the increase in emulsion viscosity (24). The differences in flow behavior of the emulsions under shear rate are well in agreement with their microstructures (Figure 1), indicating that the higher η is related to the enhanced interactions between the droplets.

The flow curves were fitted using the Carreau–Yasuda model as follows:


(2) $$\begin{array}{l} \left(\eta -\eta_{\infty }\right)/\left(\eta_{0}-\eta_{\infty }\right)={\left[1+{\left(\text{k}\gamma \right)}^{\text{a}}\right]}^{\left(\text{n}-\;1\right)/\text{a}}, \end{array}$$

where η_∞_ is the viscosity (Pa·s) at the infinitive shear rate and η_0_ represents the viscosity (Pa·s) in very low shear-rate, η stands for the apparent viscosity (Pa·s), k is the consistency index (Pa·s^n^), γ is the shear rate (s^−1^), and n represents the flow behavior index. Those parameters are summarized in Table 1. For all emulsions, the correlation coefficient *R*^2^ was > 0.99, reflecting that the Carreau-Yasuda model fitted well for the flow behavior curves. The values of flow behavior index n were low (0.16924–0.35227), demonstrating that the HIPPEs are typical non-Newtonian fluid (13). The HIPPEs with 0.5% xg/ly nps concentration showed the lowest viscosity, indicating that the emulsion exhibited more pseudoplastic behavior than those with 1–4% xg/ly nps concentration. Lower *k* and higher *n* values also verified this phenomenon. The formation of the network in the HIPPEs may be attributed to the increased particle concentration.

**Table 1:** Fitting parameters by Carreau-Yasuda model for high internal phase Pickering emulsions.

**Particle concentration**	***k***	***n***	***R*** **^2^**
0.5%	1.10857	0.35227	0.99949
1%	0.63957	0.22023	0.99985
2%	4.99302	0.16924	0.99999
4%	1.94709	0.30466	0.99997

### Viscoelastic Properties

**Figure 6:**
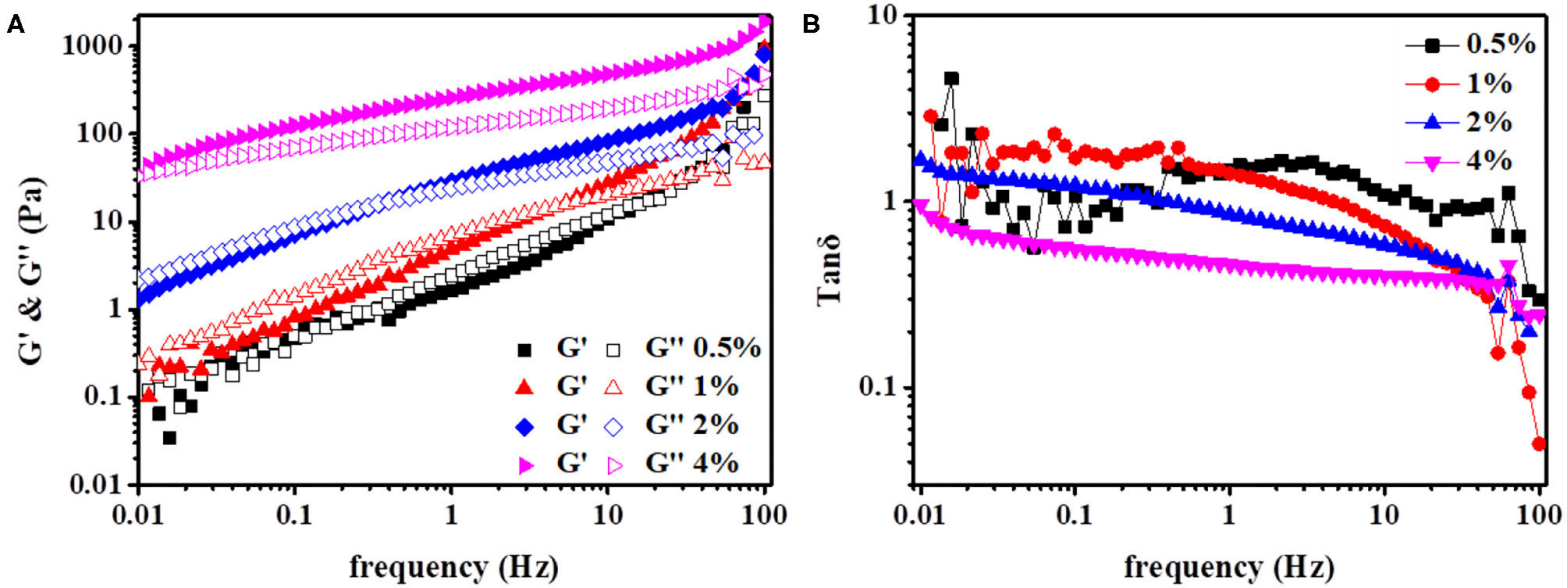
Variations of elastic (G′, solid lines) and loss (G″, dash lines) moduli **(A)**, as well as loss tangent (tan δ), **(B)** with frequency at 20°C for the high internal phase Pickering emulsions with various particle concentrations (0.5–4%).

The viscoelastic properties of the HIPPEs with various particle concentrations (0.5–4%) were further characterized using frequency sweep measurements, and the results are presented in Figure 6. As shown in Figure 6A, G′ increased obviously after the xg/ly nps concentration was added. The slopes of G′ for the emulsion of 0.5% xg/ly nps concentration were greater than that for the emulsion of 4% xg/ly nps concentration, indicating that the former was more frequency-dependent than the latter. A similar phenomenon was also found in the research of Tang et al. (21). Within the frequency range set in this experiment, the values of G′ progressively increase with particle concentration increasing from 0.5 to 4%. G′ values at a frequency of 0.1 Hz increased from 0.47 to 124.36 Pa when the particle increased from 0.5 to 4%. Meanwhile, both G′ and G″ progressively increased along with the increase of frequency, indicating that the HIPPEs turned to be a solid-like emulsion with permanent interactions behavior (23).

For emulsions with different XG/Ly NPs concentrations, the change in loss factor tan δ as a function of frequency is shown in Figure 6B. The tan δ values of all Pickering emulsions showed a slight downward trend. A similar phenomenon has also been reported in the tan δ values of Pickering emulsions stabilized by XG/Ly NPs at different XG concentrations (13). For emulsion with a low xg/ly nps concentration of 0.5%, the tan δ decreased at a small frequency. Differently, for emulsions with high xg/ly nps concentrations (1, 2, and 4%), the values of tan δ remain essentially constant in the frequency ranging from 0.01 to 46 Hz except decreasing after the frequency approaches 63 Hz. Besides, it is obvious that the network of the emulsions, except for 4% xg/ly nps concentration, exhibited more viscous properties over a large frequency range (27). However, the particle contributed more to the elastic nature of the network at high frequencies.

For all HIPPEs, G′ increased regularly with increasing frequency (Figure 6A). Combined with the theory of polymer dynamics, it is known that the frequency dependence of G′ exhibits a good power-low relationship, which can be described as G′-ω^n^. The exponent n is calculated from the logarithmic graph of G′ and ω, which can provide information for the network structure of emulsions. The exponent *n* = 0 indicates that the internal structure of the emulsion is an elastic covalent gel, and the exponent *n* > 0 indicates that the emulsion is composed of non-covalent physical crosslinking (28). The data in Table 2 showed that the *n* values of the HIPPEs stabilized by xg/ly nps fluctuate from 0.41 to 0.73 (*n* > 0). This finding suggested that the networks of the HIPPEs were constituted of non-covalent “physical” crosslinks (29). The *n* value decreased slightly with the increase in xg/ly nps concentration, indicating that improving the particle concentration contributed to the solid-like elastic properties of the HIPPEs (13). The phenomenon agrees well with the results of Figure 6B.

**Table 2:** The power-law exponent (*n*) of the frequency dependence on the storage modulus is calculated according to the equation G′-ω^n^, as a function of particle concentration.

**Particle concentration**	***k***	***n***	***R*** **^2^**
0.5%	0.64335	0.72592	0.80130
1%	1.65986	0.60249	0.92130
2%	8.74163	0.50105	0.95860
4%	118.551	0.40789	0.83380

### Large Deformation Rheology Analysis

**Figure 7:**
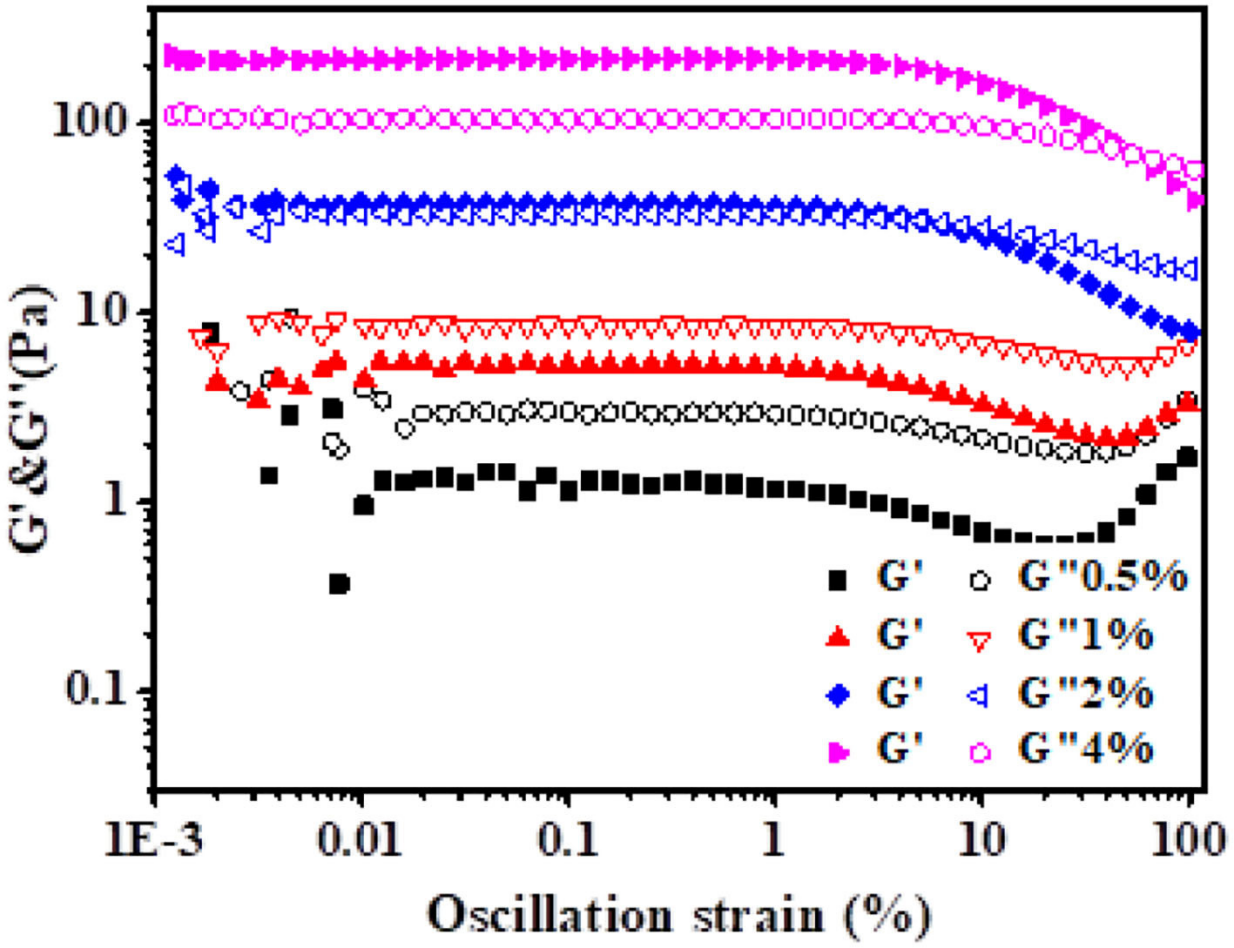
Storage modules (G′) and loss modules (G″) of high internal phase Pickering emulsions with various particle concentrations (0.5–4%) influenced by strain.

Figure 7 shows the variation of storage modulus G′ and loss modulus G″ with the oscillation strain. Within the strain range of 0.01–10%, G″ was >G′ at low xg/ly nps concentration (0.5–1%), the HIPPEs mainly exhibited viscous properties. However, an opposite trend was shown when the particle concentration was higher than 2%, indicating that the HIPPEs exhibited more elastic properties. Furthermore, as the shear strain increased, G′ decreased due to the destruction of the interdroplet structure, and G″ passed through the intersection point, γ_co_ (where G′ = G″) (30). As G″ dominated over G′ beyond this point, γ_co_ could be regarded as the mark of the HIPPEs' transition from elasticity to viscosity. A similar phenomenon occurred in the study of Reza et al. (28). For emulsions stabilized by xg/ly nps with a 2% concentration, the γ_co_ value was 6.43%, which indicated that the emulsions were monodisperse emulsions (28). When the xg/ly nps concentration was increased to 4%, the γ_co_ values increased to 53.1%, which was attributed to the shear in the inter-droplet space and/or the rheopexy of emulsions (31).

### Cryo-SEM Analysis

**Figure 8:**
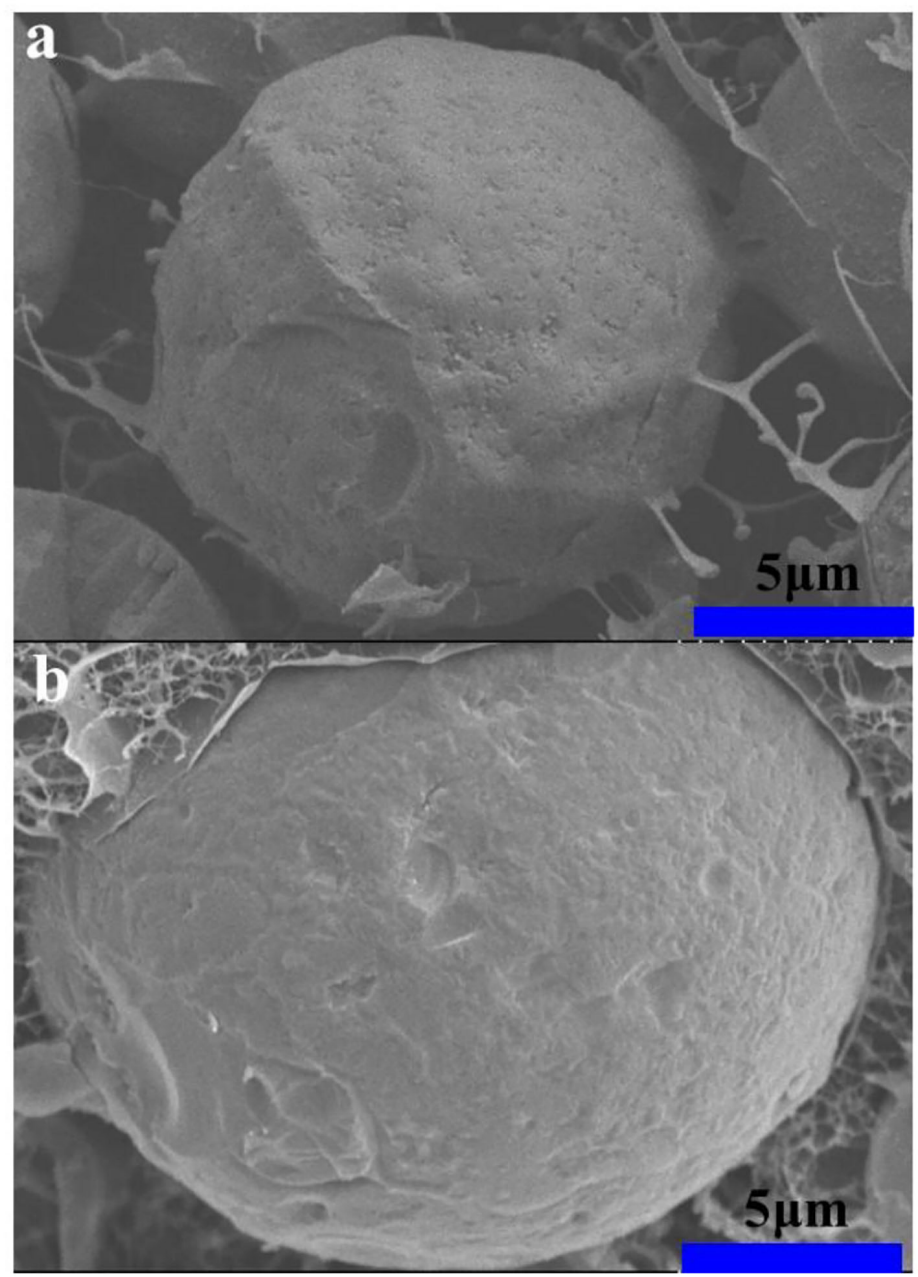
Cryo-SEM images of high internal phase Pickering emulsions stabilized with different concentrations [**(a)** 2%, **(b)** 4%, w/v] of XG/Ly NPs.

Cryo-SEM images (Figure 8) show that XG/Ly NPs existed on the oil-water interface and formed a particle adsorption layer. The surface of the Pickering emulsion droplets with a particle concentration of 2% was slightly rough and showed many small holes on the surface. These emulsions could be easy to coalesce and flocculate phenomena. It showed weaker stability than the emulsion with a higher particle concentration (4%) because the emulsion with 4% particle concentration has smooth and thick interfacial film to protect the emulsion from external forces. Meanwhile, the strong network structure formed by XG/Ly NPs in the continuous phase can prevent adjacent oil droplets from merging. The network structure provided the emulsion with more elastic properties and improved the stability of the HIPPEs. The droplet size of the emulsion was smaller than that of the emulsion with lower particle concentration, which is consistent with the results of optical microscopy (Figure 1) and CLSM images (Figure 2).

## Conclusion

A variety of novel edible HIPPEs was developed using XG/Ly NPs as stabilizers *via* a one-step emulsification process. Through the analysis of visual appearance and stability, it was found that the HIPPEs with various XG/Ly NPs concentrations (1–4%) remained stable after room-temperature standing for 3 h. The microstructure and rheology suggested that smaller droplets with increased viscosity could be formed and even the gel-like structure could be regulated by increasing the XG/Ly NPs concentration. The HIPPEs with outstanding stability regulated by XG/Ly NPs concentrations provide a simple method to fabricate HIPPEs with controllable properties. The attempt also broadens the utilization of tea oil and its application in food, cosmetics, and medicine.

## Data Availability Statement

The original contributions presented in the study are included in the article/supplementary files, further inquiries can be directed to the corresponding author/s.

## Author Contributions

WX: supervision. ZL, HL, and YL: data curation. HS: writing of the original draft. SZ: formal analysis. DL: methodology. MW: validation. YW: writing and reviewing. All authors have read and agreed to the published version of the manuscript.

## Funding

This work was financially supported by the National Natural Science Foundation of China (Grant Nos. U2004160 and 31701647), Natural Science Foundation of Henan province (Grant No. 162300410229), University Student Scientific Research Projects of Xinyang Normal University (2021-DXS-114), and Nanhu Scholars Program for Young Scholars of XYNU. This research was also kindly supported by the Analysis and Testing Center of Xinyang Normal University.

## Conflict of Interest

The authors declare that the research was conducted in the absence of any commercial or financial relationships that could be construed as a potential conflict of interest.

## Publisher's Note

All claims expressed in this article are solely those of the authors and do not necessarily represent those of their affiliated organizations, or those of the publisher, the editors and the reviewers. Any product that may be evaluated in this article, or claim that may be made by its manufacturer, is not guaranteed or endorsed by the publisher.
